# Editorial Correspondence

**Published:** 1856-11

**Authors:** J. Gotham

**Affiliations:** New York


From the Medical and Surgical Reporter.

*Editorial Correspondence.*

NEW YORK, September, 1856.

*Dull Times.*—Mr. EDITOR : "Dull Times" is still the cry of the professional men, to the great gratification of the sovereign people of this city. Salubrity is indicated by the reduced figures of the weekly bills of mortality, and by the fact that the yellow fever, which still hovers upon our borders, and sends an occasional case within, has been unable thus far to make any impression whatever.

*Ship Ahoy !*—An exception to the general dearth of occupation, however, is to be found at Quarantine, where the health officer of the port holds supreme command over as large a fleet, probably, as swelled their sails under the eye of Nelson at Trafalgar. On any day during the past two months may have been seen at anchor in our lower bay, a crowd of argosies, laden with the wealth of southern climes, detained there by the fiat of a medical officer, to whose discretion is committed the lives of the teeming thousands of this metropolis. The office has this year been no sinecure, though his income *it is said* will amount to the fabulous sum of $100,000. This is I think impossible, though the office is admitted to be the most lucrative one in the State, and can hardly be exceeded in the country. And it is right that it should be, for it is a post of exceeding great danger, and he who faithfully discharges its duties, does so to save the lives of thousands of people and millions of property, at the imminent daily risk of his own.

*Yellow Fever in and near Brooklyn.*—Our sister city of Brooklyn has not been so highly favored with health, as the yellow fever has found a pabulum there which it in vain sought for on this side the river, and it will doubtless find victims there until the appearance of frost. It is the extreme southern portion of that city only that is affected, though the district appears to extend beyond the limits of the city itself. Two physicians of New Utrecht have yielded up their lives in the performance of their duty in the epidemic ; they were Dr. Dubois, and his partner, Dr. J. L. Crane, the latter leaving a wife and several children ; and Dr. Bailey, an army surgeon stationed at Fort Hamilton, is down with it.

*Amputation at the Knee-Joint, &c., in New York Hospital.*—I was a spectator of a handsomely performed amputation at the knee-joint, one day this month, at the N. York Hospital, by Dr. Markoe. The operation was rendered necessary by a smashing of the leg of a man by a falling building ; it was doubtful for several days whether he could survive the shock of the accident, but he rallied so far as to justify the operation, under the influence of ether—but his life succumbed in twenty-two hours afterwards.

Among the curious freaks of rail-car wheels is the case of a man now in the same institution, from whose leg, from about three inches below the knee, the entire skin has been stripped, without breaking a bone or wounding a muscle, though the fascia of the latter is fully exposed. It could not have been done more neatly in the dissecting-room. The flaying process extended over the entire heel also, as far as the sole of the foot. A consultation of the surgical staff resulted in the conclusion that amputation would be necessary, as integumentary reparation is impossible when the entire circumference of the limb is denuded to so great an extent.

*Sir John Holland, and other matters.*—I observe in the daily press a notice of the arrival at Boston of Sir Sohn Holland, a distinguished English physician. He married a daughter of that famous wit, philosopher, editor, and divine, Sydney Smith. Since the visit to our shores of Marshall Hall, some two years ago, we have not been favored with the presence of any of our distinguished professional brethren of Europe, and it is rather remarkable that we have had so few of them at any time. Independent of the numbers of students and young physicians who visit Europe from this side the water, for the purpose of a prosecution of their studies abroad, we probably send on visits of pleasure to England and the Continent in one year, as many as have returned the compliment in the last ten or twenty years. To what can we attribute this contrast? It can hardly be the superior pecuniary ability of American physicians, which enables them to enjoy the pleasures of travel to so much greater extent, for England and the continent doubtless exchange with each other abundantly in this respect. We certaiuly have natural attractions sufficient to allure the medical savans of Europe from their confinement to the routine of practice, to a "ventilation" and expansion of their brains and bodies. Why then are we thus forsaken by them? Can you explain the phenomenon? I am loth to believe that the gross ignorance of our physical and political grandeur, which has been heretofore chargeable upon the politicians and the masses ot their people, but which is now fast giving way to the light of the reality, still shrouds the medical profession of Europe. Yet it may be, and if so, we can but hope that their eyes may soon be opened, and that seeing the beauty and strength of their young competitor for the glories of science, and the improvement of humanity, they may come to acknowledge us as we think we deserve, and by affording us the gratification of a more abundant personal intercourse, receive from us in return a renewal of their youth and strength. I trust the day is not far distant when at our national reunions, it will be found necessary to reserve seats for distinguished guests from abroad.

Respectfully yours,

J. GOTHAM, Jr., M. D.

